# Colloid Metals in Typhoid

**Published:** 1915-10

**Authors:** 


**Colloid Metals in Typhoid.** Maurice Villaret of Paris in Paris Medical. The solutions used containing 1/4 m.g. per c.c. Colloid silver, gold and occasionally platinum and rhodium are used. The dose is about 10 C.C. daily, administered slightly above the body temperature, subcutaneous and intra-muscular injections being as efficacious as intravenous and safer. In some cases, 10 C.C. of silver was administered along with 2 C.C. of gold. This method does not notably affect normal cases of typhoid but is especially indicated in those with septic complications. Aseptic "abscesses" sometimes occur spontaneously and may be provoked by injections of turpentine or of etherized camphorated oil and seem to favor the fixation of the infection.

